# Bu Shen Yang Xue Prescription Has Treating Effect on Endometrial Cancer through FSH/PI3K/AKT/Gankyrin/HIF-*α*/cyclinD 1 Pathway in Ishikawa Cells

**DOI:** 10.1155/2018/8412984

**Published:** 2018-10-09

**Authors:** Yue-qun Chen, Hua-li Fei, Hong-li Zhu

**Affiliations:** ^1^Department of Obstetrics and Gynecology, Affiliated Hangzhou First People's Hospital, Zhejiang University School of Medicine, Hangzhou 310006, Zhejiang Province, China; ^2^Department of Obstetrics and Gynecology, Hangzhou Women's Hospital, Hangzhou 310008, Zhejiang Province, China

## Abstract

*Background. *The formulation of Bu Shen Yang Xue (BSYX) has been clinically used in treating gynecologic disease in China, especially for the development of the endometrium. Endometrial carcinoma is the most common malignant tumor of the female genital tract in developed countries. And few studies have been reported on the antitumor activity of BSYX. Therefore, this study aimed to investigate the effect of BSYX on endometrial cancer and make an initial discussion of the underlining mechanisms in Ishikawa cells.* Methods and Results*. Firstly, 60 SPF female nude mice were randomly divided into control group, model group, BSYX group, and positive group. The models of subcutaneous tumor xenograft of nude mice were established by injection of human endometrial carcinoma cell line Ishikawa tumor cell suspension. Compared with model group, BSYX reduced effectively tumor volume and changed pathological feature in mice tumor issue. Meanwhile, proteins from tumor issues were detected by western blot analysis. The protein levels of follicle-stimulating hormone receptor (FSHR), p-Akt/Akt, Gankyrin, and cyclinD1 in the model group were higher than those in control group but the expression in BSYX group was lower than that in the model group. The hypoxia inducible factor alpha (HIF-*α*) protein level in the model group was lower than those in control group and upregulated in BSYX group. In addition, Ishikawa cells were cultured and then exposed to follicle-stimulating hormone (FSH), LY294002, a highly selective PI3K inhibitor and serum containing BSYX, respectively. LY294002 and BSYX markedly decreased the cancer cell viability and migration ability and increased the apoptosis rate. FSH promoted the cancer cell ability and migration ability. LY294002 and BSYX evidently downregulated the proteins levels of FSHR, p-Akt/Akt, Gankyrin, and cyclinD1 and upregulated the expression of HIF-*α* protein, and FSH was on the opposite.* Conclusions*. Taken together, our results showed that the formulation of BSYX had antitumor effect on endometrial cancer in vivo and in vitro and was related with FSH/PI3K/AKT/Gankyrin/HIF-*α*/cyclinD1 transduction pathway.

## 1. Introduction

Endometrial cancer, also known as uterine body cancer, refers to a group of epithelial malignant tumors that originated from the endometrium, most of which originated from the endometrial gland and it is called endometrial adenocarcinoma. Endometrial cancer is one of the three most common malignant tumors in the female reproductive tract, accounting for 8% of the total malignant tumors in the female body and 20% to 30% of the malignant tumors in the female reproductive tract [1]. In recent years, the incidence of endometrial cancer has increased significantly at home and abroad due to the prolongation of human life and the widespread use of exogenous estrogen, and the age of onset tends to be younger [2, 3]. Several risk factors have been reported to be involved in endometrial carcinoma, such as hypertension, postmenopausal status, infertility, family history, and long-term use of estrogens. In spite of tremendous advances made in the diagnosis and treatment of endometrial cancer in recent years, the treatment of advanced stages of the disease is still difficult because of the unclear pathological mechanisms. Early-stage endometrial cancer has a high survival rate, while approximately 30% of patients are diagnosed with advanced stage disease [4]. The treatment principle of endometrium is mainly surgery, supplemented by radiotherapy, chemotherapy, endocrine therapy, and biological treatment. Procedure is capable of tumor resection, but there are residual cancer tumor emboli in regional lymph node metastasis, vascular, radiotherapy, chemotherapy, and hematopoietic system leading to certain side effects on the digestive tract, and the prognosis is not ideal. Therefore, it is very important to develop treatment strategies for this disease.

Treatment with traditional Chinese medicine (TCM) can improve the effect on radiotherapy and chemotherapy in clinic and also reduce the toxic and side effects of radiotherapy and chemotherapy on digestive tract and hematopoietic system. For patients with advanced endometrial cancer or those who cannot operate, radiotherapy and chemotherapy can also be treated with TCM. TCM holds that the main pathogenic factors of endometrial cancer are related to Yin and Yang deficiency, fatigue, faint praise, dampness, and heat. Bu shen Yang xue (BSYX) prescription was derived from the works of traditional Chinese medicine named Jing yue's complete work and was made up of many herbs: Herba epimedium (Yin yang huo), dodder (Tu si zi), eucommia ulmoides (Du zhong), dogwood (Shan zhu yu), ripe radix rehmanniae (Shu di huang), salvia miltiorrhiza (Dang shen), wolfberry (Gou qi), angelica sinensis (Dang gui), paeoniae (Shao yao), and Ligusticum wallichii (chuan xiong), mixed with licorice (Gan cao) and herbs. BSYX has the characteristics of nourishing liver and kidney, invigorating qi and activating blood to collect Yin, replenishing without stagnation, nourishing without satiety and satiety, and making kidney gas abundant and decayed to heaven. BSYX has been used in treating gynecologic disease in clinic, including immune premature ovarian failure, infertility, and acted on the development of the endometrium [5–7]. So in this study, we investigated whether BSYX prescription was effective in the therapy of endometrial cancer or not.

Endometrial carcinoma is a hormone-dependent malignancy. When estrogen decreases after menopause, facilitates follicle-stimulating hormone (FSH) will increase. FSH is the core hormone in mammalian reproductive process. High FSH has large impact on women's reproductive and perimenopausal physiological pathology, and the signaling pathway downstream of FSH receptor (FSHR) has become a new research field. During human proliferation and secretion, FSHR protein is expressed in endometrial epithelial cells and interstitial cells, and the expression of secretory phase is increased. FSH may regulate endometrial function [8].

There is evidence that the phosphoinositide-3-kinase (PI3K) pathway is the most commonly activated pathway in cancer, such as human cancer cell lines [9]. More importantly, PI3K signal transduction pathway was frequently altered within endometrial carcinoma, and the activation of PI3K/AKT pathway could increase the incidence of endometrial carcinoma and lead to poorer prognosis [10, 11]. In addition, Gankyrin is a member of the family of anchor protein repeat sequences and signaling pathways can be regulated to mediate the development of cancer by the interaction of proteins with proteins [12]. Gankyrin plays an essential role in estrogen-driven and G-protein coupled receptor 30-mediated endometrial carcinoma cell proliferation via the PTEN/PI3K/AKT signaling pathway [13]. Studies showed that, after FSH binds to FSHR in ovarian cancer, PI3K/AKT pathway was activated, and the downstream gene Gankyrin expression would increase correspondingly. The expression of Gankyrin mediates the ubiquitination of hypoxia inducible factor-1*α* (HIF-1*α*) and inhibits the nucleation of HIF-1*α*, thereby reducing the binding with cyclin D1 promoter, and the expression of cyclin D1 increases correspondingly. On the other hand, Gankyrin expression inhibits PTEN expression and promotes cell proliferation [14]. Given those factors, we conducted this present study about BSYX performed antitumor effect on endometrial cancer and the relationship of FSH/ PI3K /AKT/Gankyrin/HIF-*α*/cyclinD 1 pathway in vivo and in vitro.

## 2. Materials and Methods

### 2.1. Preparation of BSYX Decoction

BSYX prescription contains eleven medicinal materials that include Herba epimedium, dodder, eucommia ulmoides, dogwood, ripe radix rehmanniae, salvia miltiorrhiza, wolfberry, angelica sinensis, paeoniae, and Ligusticum wallichii, mixed with licorice. These dried slices were acquired from Beijing Tongrentang Drugstore in China. The raw herbs were first boiled by 8-fold of water (v/w) for 2 h at atmosphere pressure. The decoction was then collected; an additional 6-fold of water was further added and boiled for 1.5h. The second decoction was separated and mixed with the first, which was then concentrated by heating at low-atmosphere pressure condition. Finally an extract of 1 mg/mL was prepared in a large scare for detailed experimental investigations.

### 2.2. Cell Culture

Human endometrial carcinoma Ishikawa cells, an estrogen-responsive cell line derived from a well-differentiated endometrioid carcinoma, were obtained from the National Infrastructure of Cell Resource, China. The cells were maintained in Dulbecco's modified Eagle's medium (DMEM high glucose) (Gibco BRL. Grand Island, NY, USA), with 10% fetal bovine serum (Gibco BRL. Grand Island, NY, USA), 100 U/ml penicillin, and 100 U/mL of streptomycin in a humidified atmosphere of 5% CO_2_ at 37°C.

### 2.3. Animal Experiments

60 SPF four-week-old female nude mice (BALB/C) were provided by zhejiang Chinese Medical University Laboratory Animal Research Center. Mice were randomly divided into control group, model group, BSYX group (2.5, 5 and 10 g/kg), and Cisplatin group, and each group had 10 mice. Besides the control group, the models of subcutaneous tumor xenograft of nude mice with human endometrial carcinoma were established by right scapular subcutaneous injection of 1 × 10^7^ Ishikawa tumor cell suspension. Tumor formation was about three weeks. Then BSYX group was treated with intragastric injection administration of BSYX,and the control group and model group were injected with normal saline for 14 days. Tumor volumes were gauged with vernier calipers regularly and the growth curves for tumors were plotted as mean ± SD of tumors from 8 mice. Tumor volume was evaluated according to the following formula: 1/2×L^2^×W (L = tumor length and W = tumor width). Antitumor rate = (C-T) /C×100% (C=the average volume of the tumor in the model group, T=the average volume of the transplanted tumor in the treatment group).

The mice were housed in a specific pathogen-free environment suitable for survival and were observed and measured on a regular basis. Later the mice were sacrificed and the tumors excised to compare the size of the tumor. All mouse experiments were approved by the animal ethics committee of Zhejiang University.

### 2.4. Hematoxylin-Eosin (HE) Staining

The tumor tissues were immersion-fixed in 10% neutral buffered formaldehyde solution and embedded in paraffin for a light microscopic study. The sections of formalin-fixed, paraffin-embedded tissue samples were deparaffinized in xylene and rehydrated in graded ethanols. The specimens were routinely processed and stained with HE for histopathological classification.

### 2.5. The Serum Containing BSYX Preparation

The ICR female mice weighing 18–22 g were randomly divided into following groups (n=10): a control group (normal saline); three BSYX groups (2.5g/kg, 5g/kg, and 10 g/kg). The control saline or BSYX groups were administered via intragastric injection every 2 days for five times. At day 14, all the animals were sacrificed and the drug serum and the blank serum were separated from heart blood. The blood was centrifuged at 4000 rpm for 10 min at 4°C to obtain serum. The serum was labeled and stored at −70°C.

### 2.6. Cell Treatment and Grouping

Ishikawa cells were cultured and then divided into six groups: normal control group, FSH (50 mIU/ml), LY294002 (15*μ*mol/L), blank serum, and drug serum from mice treated BSYX (2.5, 5, and 10 mg/kg). The concentrations of FSH and LY294002 were selected by reference to relevant literatures about endometrial cancer cells. [15, 16]

### 2.7. CCK8 Assay

The CCK8 assay was performed using a CCK8 kit following the manufacturer's protocol (Dojindo, Kumamoto, Japan). Briefly, cells were plated into 96-well plates (2 × 10^3^ cells per well) in 100 *μ*L of culture medium and incubated for 24 h at 37°C. CCK-8 solution (100 *μ*L/well) was added for another 2 h and then incubated for 24 h. Before reading the plate, it is important to mix gently on an orbital shaker for 1 minute to ensure homogeneous distribution of color. Then, the absorbance was measured at 450 nm with a Thermomax microplate reader (Molecular Devices, Sunnyvale, CA, USA**)**. Each experiment was repeated three times.

### 2.8. Annexin V-FITC/PI Staining

Cell apoptosis/necrosis was evaluated using Annexin V-FITC/ PI staining. Cells were plated into 6-well plates. After treatment for 24 h, cells were collected and washed twice with cold phosphate-buffered saline (PBS). The cells were then resuspended in 500 *μ*L buffer with 100 *μ*L of buffer and 5 *μ*L Annexin V- FITC and 5 *μ*L propidium iodide (PI) and incubated for 15 min in the dark at room temperature. Binding buffer (250 *μ*L) was added to each tube, and the apoptosis rate was determined by flow cytometry within 1 h. Annexin V−/PI−cells are viable, annexin V+/PI− cells are in early apoptosis, and annexin V+/PI+ cells are necrotic or in late apoptosis

### 2.9. Cell Migration Rate Detection

Cells were seeded in 6-well plates at the density of 5 × 10^5^cells and cultured until confluence. The cell monolayer was scratched with a sterile pipette yellow tip to produce a straight line, and the debris was washed out with culture medium. Medium and substances for treatment were renovated at 24 h or 48 h. The open gap was photographed with a camera at phase contrast microscope at the indicated times. The rate of healing was estimated by ImageJ software based on the area (in which the length is predetermined and the wideness varies) free of cells. Each determination represents the average of 3 individual experiments.

### 2.10. Western Blot

The total proteins from cell or tumor issue were harvested and lysed in RIPA buffer containing 1 mM phenyl methane sul-fonyl fluoride (Beyotime Biotechnology, Shanghai, China) and mixed with loading buffer. Protein concentration was determined by BCA kit (Beyotime Biotechnology, Shanghai, China). Proteins (10*μ*g per lane) were separated by sodium dodecylsulfate polyacrylamide gel electrophoresis and then transferred to a polyvinylidene difluoride membrane (PVDF, Millipore, Billerica, MA, USA). The membranes were blocked with PBS containing 5% skim milk for 2 h and then incubated overnight with primary antibodies against Bax (diluted at 1:1000; #5023, CST), Bcl-2 (diluted at 1:1000; #4223, CST) AKT (diluted at 1:1000; #4691, CST), phospho (p)-AKT (diluted at 1:1000; #4060, CST), FSHR (diluted at 1:500; ab75200, abcam), Gankyrin (diluted at1:500;#12985, CST), HIF-*α*(diluted at 1:500; ab179483, abcam), cyclin D1(diluted at 1:500;#2978, CST), and GAPDH (diluted at 1:1000; #5174, CST) at 4°C. The membranes were washed three times with TBST (Tris-buffered saline containing Tween-20) and then anti-rabbit secondary antibodies (1:5000) were added. Following 2 h incubation at room temperature, the protein bands were visualized by enhanced chemiluminescence (ECL, Beyotime Biotechnology, Shanghai, China) according to the manufacturer's instruction. The images were obtained using Mini-PROTEAN gel imaging system. GAPDH was served as a loading control, and protein expression was quantified by optical density obtained using ImageJ software program.

### 2.11. Statistical Analysis

The experimental data were analyzed using SPSS 19.0 software. Comparisons among multiple groups were made with one-way analysis of variance followed by Fisher's least significant difference test, and all data were the mean ± standard deviation of at least three separate experiments;* P*<0.05 was considered statistically significant.

## 3. Results

### 3.1. Effect of BSYX on Tumor Volume and Histomorphology in Mice

**Figure 1:**
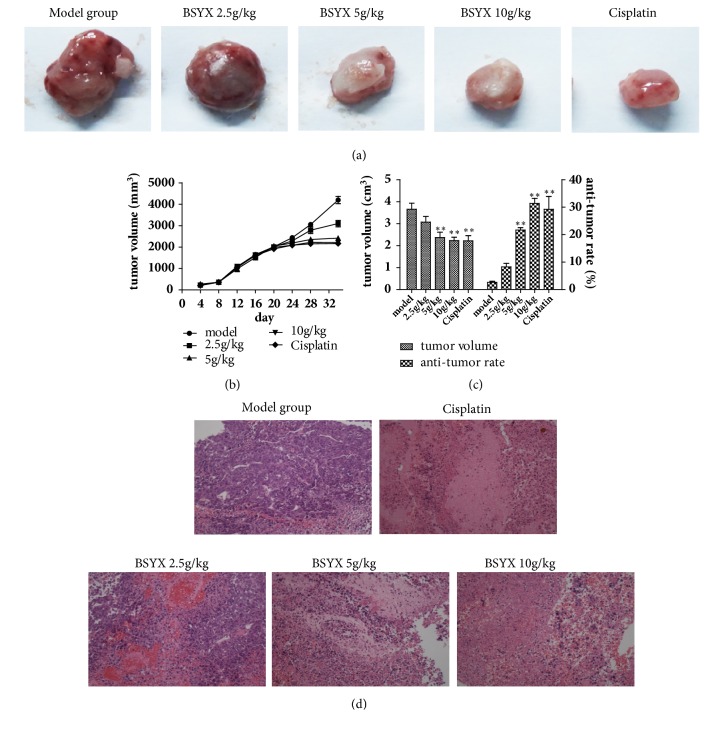
Effect of BSYX on Tumor volume and histomorphology in mice tumor issue. After treating for 14 days, tumors from mice were collected (a), growth curve of tumors was obtained from tumor measure every four days (b), and the tumor volume and antitumor rate (c) were calculated. The tumor issue was observed with stained with H&E (magnification of all figures ×200) (d). *∗∗*P<0.01 versus the model group.

After treatment for 14 days, we collected the tumor and calculated tumor volumes and the anti-tumor rates. As shown in Figures 1(a) and 1(b), the xenograft model of human endometrial carcinoma in nude mice was successfully prepared. The tumor volumes and antitumor rates in the treating groups treated with BSYX(5 and 10 g/kg) and cisplatin were reduced compared with model group(P<0.01) (Figure 1(c)). H&E staining was shown in tumor tissue (Figure 1(d)). The tumor cells in the model group were closely arranged, with lots and different sizes of cancer cells or cell nucleus, less interstitium, and asymmetrical pathological nuclear division phases. In BSYX 2.5g/kg group, tumor cells are dense and irregularly arranged, with large nuclei, deep staining, and numerous mitotic figures. In BSYX 5 and 10 g/kg group tumor cells are arranged loosely, with regular morphology and fewer mitotic figures, and the BSYX 10 g/kg group was the better.

### 3.2. Effect of BSYX on Levels of AKT, p-AKT, FSHR, Gankyrin, HIF-*α*, and Cyclin D1 Proteins in Mice Tumor

**Figure 2:**
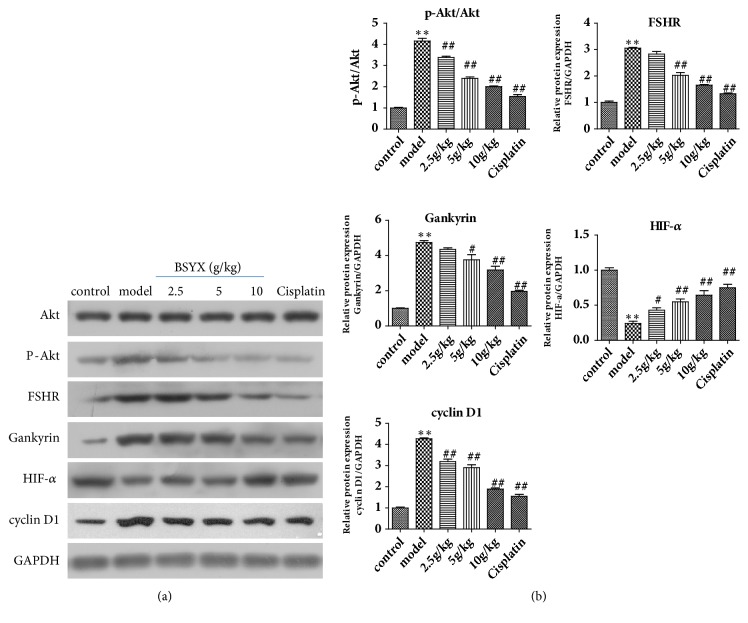
Effect of BSYX on levels of AKT, p-Akt, FSHR, Gankyrin, HIF-a, and cyclin D1 proteins in mice tumor. AKT, p-AKT, FSHR, Gankyrin, HIF-a, and cyclin D1 proteins levels were measured with Western blotting and normalized to GAPDH (a). Relative band intensities were used in order to quantify AKT, p-Akt, FSHR, Gankyrin, HIF-*α*, and cyclin D1 protein (b) expression levels. Data are expressed as the mean ± S.E.M. n = 10 mice in each group. *∗∗*P<0.01 versus the control group, ^#^P<0.05, and ^##^P<0.01 versus the model group.

The Akt pathway has been frequently reported in cancer, so we examined Akt phosphorylation status. The results showed an evident increase of p-Akt/Akt in model group (*P*<0.01). Compared with the model group, p-Akt/Akt protein expression was reduced in the treating groups treated with different dose of BSYX and cisplatin (*P*<0.01). FSHR and Gankyrin/HIF-*α*/cyclinD1 were upstream and downstream genes of P13K/Akt signal pathway, respectively, then their proteins expression levels were detected. As shown in Figures 2(a) and 2(b), in model group, the levels of FSHR, Gankyrin and cyclinD1 were distinctly increased (*P*<0.01) and the levels of HIF-*α* were distinctly decreased (*P*<0.01). Compared with the model group, the levels of FSHR, Gankyrin, and cyclinD1 in BSYX group were distinctly decreased (*P*<0.01), and the levels of HIF-*α* were distinctly increased (*P*<0.01) in a concentration dependent manner.

### 3.3. Effect of BSYX on Cell Ability and Apoptosis Rate in Human Endometrial Carcinoma Ishikawa Cells

**Figure 3:**
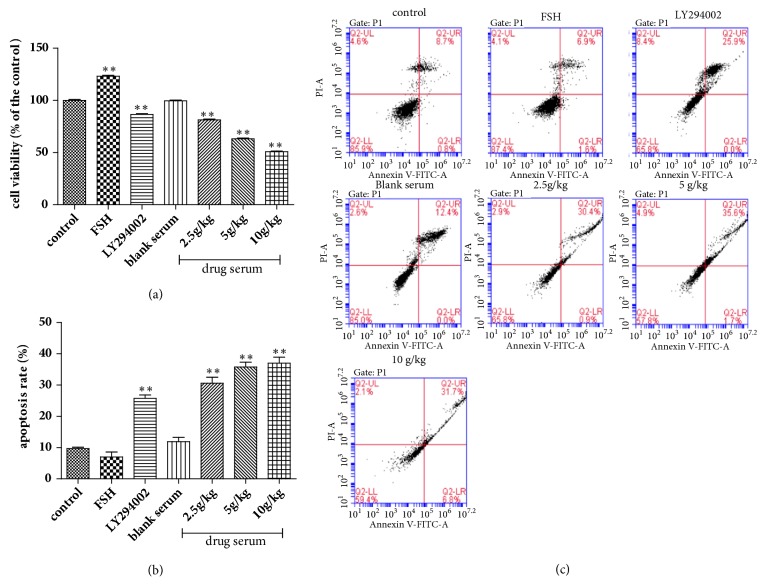
Effect of BSYX on cell ability and apoptosis rate in human endometrial carcinoma Ishikawa cells. Cells were treated with FSH, LY284002, blank serum and drug serum containing BSYX for 24 h, then cell ability was detected by CCK8 assay and was calculated (a). Cells were double stained with annexin V-FITC and PI and analyzed by flow cytometry. And percentage of apoptotic cells was shown by histogram ((b) and (c)). Data are expressed as the mean ±SEM n = 3 in each group. *∗*P<0.05,*∗∗*P<0.01 versus the control group.

**Figure 4:**
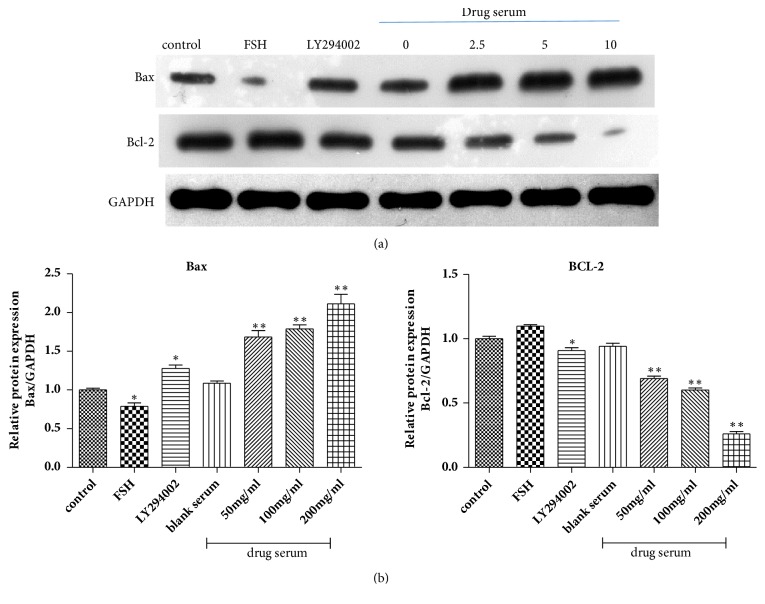
Effect of BSYX on levels of Bax and Bcl-2 proteins in human endometrial carcinoma Ishikawa cells. Bax and Bcl-2 proteins levels were measured with Western blotting and normalized to GAPDH (a). Relative band intensities were used in order to quantify Bax and Bcl-2 proteins (b) expression levels. Data are expressed as the mean ± SEM n = 3 in each group. *∗*P<0.05,*∗∗*P<0.01 versus the control group.

Human endometrial carcinoma Ishikawa cells were stimulated for 24 h with serum of BSYX in a gradient concentration, FSH and LY294002, respectively. Cell ability was detected by CCK8 assay. As shown in Figure 3(a), compared with control group, FSH improved the tumor cell viability (*P*<0.01), and LY294002 reduced the tumor cell viability (*P*<0.01), cell viability was decreased significantly with increasing BSYX concentrations (*P*<0.01). Cell death after treatment with BSYX was also measured using flow cytometric analysis with Annexin V-FITC/PI staining. As shown in Figure 3(b), compared with control group, LY294002 promoted the tumor cell apoptosis, which was about 25.78 ± 1.04%(*P*<0.01), cell apoptosis rate was increased significantly in BSYX group(*P*<0.01) and was also higher than the LY294002 group. In addition, the proapoptosis protein Bax and the antiapoptosis BCl-2 were observed. As shown in Figure 4, compared with control group, FSH decreased the levels of Bax protein expression (*P*<0.05) and increased the levels of Bcl-2, LY294002 and BSYX increased the levels of Bax protein expression and decreased the levels of Bcl-2 (P<0.05, P<0.01),

### 3.4. Effect of BSYX on Migration Ability in Human Endometrial Carcinoma Ishikawa Cells

**Figure 5:**
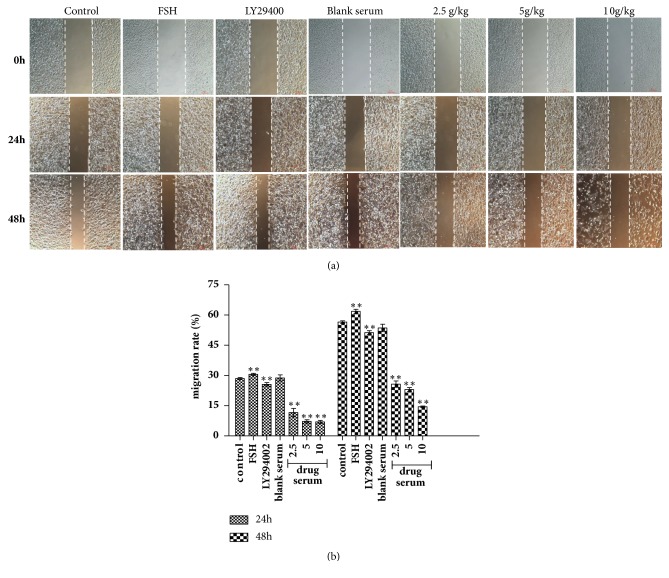
Effect of BSYX on migration ability in human endometrial carcinoma Ishikawa cells After BSYX treatment, the cell migration ability was analyzed by the wound healing assays (a) and the migration rate was calculated (b). Data are expressed as the mean ± SEM n = 3 in each group. *∗*P<0.05,*∗∗*P<0.01 versus the control group.

Ishikawa cells were stimulated for 24 h or 48 h with serum of BSYX in a gradient concentration. As shown in Figure 5, compared with control group, FSH increased the tumor cell migration ability (*P*<0.01), LY294002 decreased the tumor cell migration ability, which were about 25.62 ± 0.83% and 51.29 ± 0.94%, respectively, at 24 and 48 h(*P*<0.01). Cell migration ability in BSYX groups were decreased significantly and lower than those in the control and LY294002 groups (*P*<0.01).

### 3.5. FSH/AKT/Gankyrin/HIF-*α*/CyclinD 1 Pathway Plays an Important Role in BSYX Impact on Human Endometrial Carcinoma Ishikawa Cells

**Figure 6:**
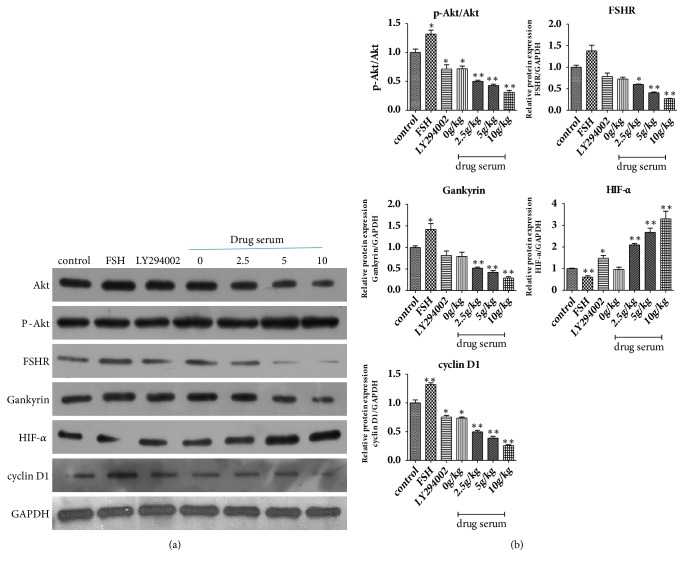
Effect of BSYX on levels of AKT, p-Akt, FSHR, Gankyrin, HIF-a, and cyclin D1 proteins in human endometrial carcinoma Ishikawa cells. AKT, p-Akt, FSHR, Gankyrin, HIF-*α*, and cyclin D1 proteins levels were measured with Western blotting and normalized to GAPDH(a). Relative band intensities were used in order to quantify AKT, p-AKT, FSHR, Gankyrin, HIF-*α*, and cyclin D1 protein (b) expression levels. Data are expressed as the mean ±SEM n = 3 in each group. *∗*P<0.05,*∗∗*P<0.01 versus the control group.

After Ishikawa cells were stimulated for 24 h with serum of BSYX in a gradient concentration, AKT, p-AKT, FSHR, Gankyrin, HIF-a, and cyclin D1 proteins were detected by western blot analysis. As shown in Figure 6, compare with control group, FSH increased the levels of p-AKT/AKT, FSHR, Gankyrin, and cyclinD1 proteins expression (*P*<0.05,* P*<0.01) and decreased the levels of HIF-*α* (*P*<0.01), LY294002 decreased the levels of p-AKT/AKT, FSHR, Gankyrin, and cyclinD1 proteins expression (P<0.05, P<0.01) and increased the levels of HIF-*α* (*P*<0.01), the levels of p-AKT/AKT, FSHR, Gankyrin, and cyclinD1 proteins expression were decreased significantly, and the levels of HIF-*α* were increased with increasing BSYX concentrations (*P*<0.01).

## 4. Discussion

The incidence of endometrial cancer is increasing year by year and it is the second leading cause of deaths due to gynaecological cancer worldwide. Recent evidence showed that TCM has great potential in treating endometrial cancer including Qingrejiedu Lianhua soup [17], astragalus polysaccharides [18], and Lithospermum [19]. In our experiment, BSYX prescription, as the classic prescription for treating gynecological diseases in TCM, was chosen to explore its effect on endometrial cancer, Firstly, according to the body surface area ratio of mice to human beings, the dosage conversion coefficient for mice to human beings is 9.1, thus, the dose ranges of BSYX in a 60-kg person are equivalent to 3–10g/kg. In this study, the doses of 2.5, 5, and 10 g/kg for 14 days were used in the treatment of the mice. The models of subcutaneous tumor xenograft of nude mice were established by injection of human endometrial carcinoma cell line Ishikawa tumor cell suspension. Our results indicated that the tumor volume in 5 and 10 g/kg BSYX group was lower while the inhibition rate was higher than those in the model group, consistent with the change in pathological morphology.

The endometrium is now considered to be two kinds of pathogenesis: one is a kind of estrogen dependent; namely, endometrial hyperplasia or even cancer may occur under the long-term action of estrogen without progesterone antagonism. This type accounts for the majority of endometrial cancer. The other is nonestrogen-dependent and has no definite relationship with estrogen [20]. The effect of FSH on target organs is mainly mediated by receptor FSHR. FSHR is not only expressed in female ovarian granule cells and male testicular cells, but also in prostate [21], ovarian epithelium [8], and other tissues. Aberrant FSHR function facilitates the development of cancer, and FSH promotes proliferation of ovarian cells [14]. PI3K/Akt pathway can be regulated by FSHR, and PI3K/Akt pathway activation was associated with enhanced invasive and migratory capacities. Inhibiting the PI3K/Akt pathway reversed progestin resistance in endometrial cancer [22]. Meanwhile, Gankyrin is crucial for breast cancer metastasis and highlight the potential of Gankyrin as a therapeutic target for tumor metastasis [23]. What is more, high expression level of Gankyrin increased hepatocellular carcinoma cell migration and invasion by PI3K/Akt pathway [24]. In addition, Gankyrin could induce cyclin D1 expression [25]. Cyclins play key roles in cell proliferation and growth. Cyclin D1 is an important mediator of cancer initiation, development, and metastasis and is associated with poor prognosis. LncRNA ABHD11-AS1 promotes the development of endometrial carcinoma by targeting cyclin D1[26]. Interfering with cyclin D1 leads to PI3K/Akt signal suppression [27]. So the signal transduction pathway of FSH, PI3K/Akt, and Gankyrin/HIF-*α*/cyclinD1 in endometrial carcinoma attracted our interest. Our results found that BSYX induced an increase in the expression levels of FSHR, Akt/p-Akt, Gankyrin, and cyclin D1 proteins and a decrease in HIF-*α* in mice tumor. Therefore the relationship of endometrial carcinoma and the FSH/PI3K/AKT/Gankyrin/HIF-*α*/cyclinD1 pathway was further discussed with BSYX intervention in vitro.

LY294002, a highly selective PI3K inhibitor, was used to block PI3K-dependent Akt phosphorylation. When exposed Ishikawa cells to FSH and LY294002, LY294002 reduced the tumor cell viability, increased cell apoptosis rate and proapoptosis Bax protein expression, decreased the anti-apoptosis Bcl-2 protein expression and the tumor cell migration ability, downregulated the levels of p-AKT/Akt, FSHR, Gankyrin, and cyclinD1 proteins expression, and upregulated the levels of HIF-*α*. However, FSH increased the levels of FSHR, p-AKT/Akt, Gankyrin, and cyclinD1 proteins expression and decreased the levels of HIF-*α*. The results obtained from exposed Ishikawa cells to BSYX were in line with LY294002. BSYX led a decrease in the tumor cell viability and cell migration ability and changed the expression levels of p-AKT/Akt, FSHR, Gankyrin and cyclinD1 and proteins HIF-*α* which were in line with those in vivo. In the next step, we plan to explore the specific components of BSYX prescription in combination with antiendometrial cancer and the mechanism of action through network pharmacology in order to provide powerful new knowledge to guide its clinical usage.

## 5. Conclusions

To sum up, 5 and 10 g/kg BSYX reduced tumor volume, changed pathological feature in mice tumor issue, also markedly reduced the human endometrial carcinoma Ishikawa cell growth ability and migration ability, and accelerated the apoptosis. The formulation of BSYX had treating effect on endometrial cancer and was related with FSH/PI3K/AKT/Gankyrin/HIF-*α*/cyclinD1 pathway, which can supply the experimental basement of its clinical usage.

## Data Availability

The data used to support the findings of this study are included within the article.
